# Supplementary material for: Are carbon emissions trading and green financial instruments synergistic? -Comprehensive quantitative research based on content analysis
Source: PLoS One. 2024 Mar 7;19(3):e0298601. doi: 10.1371/journal.pone.0298601 (PMC10919601; doi:10.1371/journal.pone.0298601)
Supplement: S1 Data — (ZIP) [file pone.0298601.s001.zip › Data/地级市Z_F工作报告.zip!/╜¡╦╒/─╧╛⌐╩╨/└┤╫╘╣1⁄2╓┌║┼í╛┬φ┐╦╩2╛▌═°í┐_2017.docx]

各位代表：

现在，我代表南京市人民政府，向大会报告工作，请予审议，并请政协委员和其他列席人员提出意见。

一、2016年工作回顾

过去的一年，面对复杂多变的宏观经济环境，我们在以习近平同志为核心的党中央坚强领导下，认真贯彻落实党中央国务院、省委省政府决策部署和市委工作要求，围绕“强富美高”新南京建设目标，坚持稳中求进工作总基调，自觉践行新发展理念，主动适应经济发展新常态，以供给侧结构性改革为主线，统筹做好改革发展稳定各项工作，较好地完成了市十五届人大四次会议确定的主要目标任务，实现了“十三五”发展的良好开局。预计全年实现地区生产总值10450亿元、增长8%，人均地区生产总值达12.65万元；一般公共预算收入完成1142.6亿元、增长12%；全社会研发经费投入316.6亿元，占地区生产总值比重达3.03%；全体居民人均可支配收入增长8.3%左右；PM2.5浓度较上年下降15.4%，单位地区生产总值能耗降幅、主要污染物排放强度达到省定标准；民生十件实事全面完成。

（一）落实五大任务、分类精准施策，经济平稳健康运行。积极应对经济下行压力，加强宏观形势研判，强化经济组织运行，着力解决制约经济稳定增长的突出问题。有针对性制定出台扩大消费、促进投资、稳定外贸等方面政策措施，切实增强经济增长协同拉动力。全力推进216个市级重大项目建设，台积电晶圆厂、中航南京机电科技研发生产基地等一批重大项目进展顺利。紧扣供给侧结构性改革目标，推动“三去一降一补”任务落地见效，全市39家企业涉及低效产能淘汰工作全部完成，五大高耗能行业产值同比下降4%左右；加大房地产市场调控力度，保持房地产健康稳定发展；置换政府债务589亿元，市政府分五年出资200亿元，设立总规模500亿元的市产业发展基金和500亿元的市城建发展基金，新增挂牌上市公司114家；落实降成本“双二十条”意见，为实体经济企业降低成本超过200亿元，规上工业企业财务费用同比下降10%左右；七大短板领域120个项目加快实施，基础设施领域投资增幅达10%。组建市企业服务中心和投资建设代办服务中心，服务实体经济特别是小微企业力度进一步加大。

（二）实施创新驱动、加快转型步伐，产业竞争力稳步提升。制定贯彻国家创新驱动发展战略纲要实施方案，出台36条政策措施，推动科技与产业紧密融合。新增高淳、麒麟、白马三家省级高新区，苏南国家自主创新示范区“一区两园多载体”联动格局初步形成。全市高新技术企业达到1500家，产值达到8800亿元、增长8.5%。积极推动大众创业万众创新，新增科技部备案众创空间27家，新增60人入选第二批国家“万人计划”，在宁高校就地转化应用技术成果1864项。实施青年大学生创业引领计划和高校毕业生租房补贴政策，培育自主创业者1.6万人，在宁高校毕业生就地就业达5.5万人。现代服务业发展步伐加快，电子商务交易额增长25%，软件和信息服务业、旅游业收入分别增长15%和13%，金融业增加值增长14%，服务业增加值占地区生产总值比重达到58.4%，较上年提高1.1个百分点。战略性新兴产业提速发展，七大类14个重点领域新兴产业实现主营业务收入6800亿元，增长13%，较上年提高2.3个百分点，其中新能源汽车、卫星应用、集成电路及专用设备等产业主营业务收入增幅超过20%。新增设施农业4.2万亩、高标准农田10万亩，农业基本现代化水平居全省第一。

（三）全面深化改革、着力扩大开放，动力活力明显增强。扎实推进各项改革，全市42项306条改革任务全部达到序时进度，21项国家和省级重点改革试点任务加快落实，行政审批、医疗卫生、户籍制度、内贸流通等改革取得明显成效。积极推进“放管服”改革，动态调整455项行政权力事项。“三位一体”国企监督体系创新列入全国国企改革50例。商事制度改革持续推进，率先实行“多证合一、一照一码”，新增市场主体、注册资本分别增长28.6%、58.7%。成立全国首家综合性文化金融服务中心，苏宁银行获批筹建江苏首家民营银行。基本完成农村土地承包经营权确权登记，农村产权交易市场实现区、街全覆盖。积极创建国家信用示范城市，正式启用市民诚信卡。持续扩大对外开放，实际利用外资34.5亿美元，跨境电子商务产业园等项目建成运营，空港经济开发区成功获批筹建省级开发区，中欧货运班列开通运行。宁镇扬一体化、南京都市圈合作务实推进，对口支援工作成效显著。

（四）加大统筹力度、完善功能品质，承载能力不断提高。南京市城市总体规划和江北新区城市总体规划正式获批，城市规划建设管理进一步加强。江北新区建设框架基本拉开，中心区基础设施建设和中央商务区地下空间开发加快推进，扬子江隧道建成通车，过江隧道免费通行。三大枢纽经济区、江心洲生态科技岛、麒麟科创园形象初显，鼓楼滨江、铁心桥—西善桥等五大片区改造顺利推进。基础设施建设步伐加快，地铁4号线一期试运行，宁和线一期、宁高线二期、宁溧线加快推进，长江五桥开工建设，红山南路东延一期等工程顺利建成。推进城市精细化管理，落实“门前三包”责任制，实施交通枢纽、特色景区及明城墙等夜景照明提升工程，推进19条干道和4个重点片区环境综合整治，拆除违规户外广告460处。实施美丽乡村示范区建设340平方公里，高淳桠溪镇成功入选第一批国家级特色小镇。

（五）加强环境治理、强化生态保护，人居质量持续改善。贯彻落实中央环保督察组反馈意见，整改一批矛盾突出的环境问题。以市政府1号文件出台《水污染防治行动计划》，下力气整治城市黑臭河道，43条黑臭河道水质明显改善，完成排水达标区创建230个，县级以上主要集中式饮用水源地达标率保持100%。强化畜禽养殖环境管理，基本完成禁养区内规模养殖场关闭搬迁。深化大气污染防治，实施125项大气污染治理重点工程和冬春季管控措施，完成30家重点企业挥发性有机物治理项目，实行黄标车和无标车全市域全时段禁行，全面执行国V排放标准。全市空气质量达到二级标准以上天数比例较上年提升1.6个百分点，其中优秀天数同比增加21天。四大片区工业布局调整取得新进展，实施100个重点节能项目，燃煤消耗下降率实现年度目标。全市绿化造林2.7万亩。南京荣获“国家生态市”称号。

（六）坚持以人为本、突出发展惠民，社会建设全面加强。持续加大民生投入力度，全市财政一般公共预算支出中民生支出占比达77.8%。精准帮扶就业创业，新增城镇就业21.52万人，城镇登记失业率控制在1.88%。全面开展新一轮农村脱贫致富奔小康行动，20%的低收入农户和经济薄弱村实现脱贫。社会保障水平稳步提升，在全省率先实现城镇职工五大险种市级统筹，城乡最低生活保障、居民基础养老金标准继续提高。各项社会事业加快发展，教育质量不断提升，义务教育优质资源覆盖率达85%；医疗健康资源布局进一步优化，市儿童医院河西院区、公共卫生医疗中心投入运行；文化惠民“百千万工程”成功创建国家公共文化服务体系示范项目。住房保障力度不断加大，新开工建设保障房495万平方米，竣工413万平方米，完成棚户区改造365万平方米，率先将公租房货币化保障对象扩大到城市中等偏下收入住房困难家庭和新市民，发放住房租赁补贴5.28亿元。公交都市建设持续推进，新辟、调整公交线路81条，新增公共自行车服务点700个，投放公共自行车近3.5万辆，累计超过7万辆，主城区公交机动化出行分担率达63%。成功举办2016年世界速度轮滑锦标赛和南京马拉松赛。深入推进平安南京、法治南京建设，实施“平安保民十项举措”，深化全国质量强市示范城市创建，全市安全生产、食品药品安全、信访形势稳定向好。再次获得“全国双拥模范城”殊荣，实现“八连冠”。特别是面对严峻汛情，全市上下一心，军民团结奋斗，取得防汛抗灾的全面胜利。全年办理市人大代表建议325件、市政协提案584件，满意和基本满意率达98.8%。社会科学、妇女儿童、残疾人、红十字、慈善事业等稳步发展，民族宗教、外事侨务、档案方志、民防消防等工作进一步加强。

各位代表，过去一年的成绩来之不易。这是党中央国务院、省委省政府和市委坚强领导的结果，是市人大、市政协和社会各界大力支持和有效监督的结果，是全市人民攻坚克难、共同奋斗的结果。在此，我代表南京市人民政府，向在各行各业辛勤劳动的全体市民，向人大代表、政协委员、各民主党派、工商联和无党派人士、各人民团体、社会各界人士和老同志，向驻宁人民解放军、武警官兵和人民警察，向所有支持南京建设与发展的香港特别行政区同胞、澳门特别行政区同胞、台湾同胞、海外侨胞和国际友人，表示衷心感谢！

我们也清醒地认识到，全市经济社会发展中还存在不少矛盾和问题：固定资产投资增长乏力，特别是工业投资出现下滑趋势，外贸出口低位运行，新的经济增长点有待加速培育；科技创新带动作用还不显著，企业创新主体地位还不突出，科技成果转化率有待提高；实体经济困难较多，中小企业融资难问题仍然存在；生态环境质量尚未根本好转，空气污染防治、黑臭河道和积淹水片区治理任务艰巨；城乡居民持续增收难度加大，公共服务资源配置仍不够均衡，交通、养老、医疗、教育等方面还存在不少薄弱环节；政府职能转变步伐还需进一步加快，依法行政意识和能力有待增强，一些政府工作人员服务意识不强，“四风”和腐败问题仍有反复。我们必须直面问题，采取更加有力措施切实加以解决，让全市人民感受到实实在在的变化和成效。

二、2017年总体思路和目标任务

今年是党的十九大召开之年、供给侧结构性改革的深化之年，也是全面落实省第十三次党代会、市第十四次党代会精神的开局之年。做好今年政府工作，要全面贯彻党的十八大和十八届三中四中五中六中全会精神，深入贯彻习近平总书记系列重要讲话特别是视察江苏重要讲话精神，按照省第十三次党代会和市第十四次党代会部署，以及省委常委会对南京发展的新要求，统筹推进“五位一体”总体布局，协调推进“四个全面”战略布局，围绕省党代会确立的“两聚一高”主题和市党代会确定的“一个高水平建成、六个显著”目标，坚持稳中求进工作总基调，牢固树立和贯彻落实新发展理念，主动适应把握引领经济发展新常态，坚持以提高发展质量和效益为中心，以推进供给侧结构性改革为主线，全面做好稳增长、促改革、调结构、惠民生、优生态、防风险各项工作，确保经济平稳健康发展和社会和谐稳定，在高水平全面建成小康社会、加快建设“强富美高”新南京上迈出坚实步伐，以优异成绩迎接党的十九大胜利召开。

今年全市经济社会发展主要指标，分为两大类：一是预期性指标。地区生产总值增长8%左右，一般公共预算收入同口径增长8%，固定资产投资增长5%以上，社会消费品零售总额增长10%；城镇新增就业16万人，城镇登记失业率控制在3.5%以内；全体居民人均可支配收入增长8%以上，其中农村居民人均可支配收入增速高于城镇居民人均可支配收入增速。二是约束性指标。单位地区生产总值能耗下降4%，燃煤消耗总量减少100万吨，控制在2980万吨以内；主要污染物排放指标完成省定任务，基本消除建成区109条河道黑臭现象，PM2.5浓度比2013年下降20%以上，空气质量达到二级标准以上天数力争达到70%。

根据省委省政府决策部署和市委要求，重点做好十个方面工作。

（一）优化供给扩大需求，全力保持经济稳定增长。进一步加大供给侧结构性改革力度，适度扩大总需求，努力实现供求关系新的动态平衡，确保经济运行在合理区间。

深入推进“三去一降一补”。实施“三高两低”企业整治，化解水泥产能150万吨、船舶产能20万载重吨，推动梅钢、南钢粗钢去产能工作。落实稳控房价措施，扩大供地规模，加强市场监管，稳定市场预期，保持房地产市场平稳健康发展。进一步降低杠杆水平，加大股权融资力度，新增挂牌上市企业60家以上。强化互联网金融风险专项整治，做好政府债务置换工作，切实防范化解可能出现的金融风险隐患。更大力度为企业降本减负，落实好国家、省市减税清费各项政策，全年为实体经济企业直接减负200亿元左右。持续聚焦基础设施、民生保障、精准扶贫等七大领域37项补短板重点任务，增强发展的整体性和协调性。

切实扩大有效需求。推动供给与需求有效对接、投资与消费有机结合、内需与外需协调拉动，筑牢支撑经济持续稳定增长的基础。全面实施新消费引领三年行动计划，积极培育服务消费、信息消费、绿色消费、品质消费等新增长点，推进全域旅游，促进消费提质升级。深入推进“互联网+商贸”，支持电子商务平台创新和模式创新，加快建设国家电子商务示范城市。持续扩大有效投入，加大科技创新、高端产业、技术改造、生态环保、公共服务等领域投资。建立央企重大项目协调推进机制，全力推进市级重大项目建设。增强外贸竞争新优势，坚持增加总量和提升质量并重，加大外贸新业态支持力度，推进跨境电子商务发展，加速服务贸易和服务外包增长，做强一批外贸综合服务企业，培育一批新的外贸增长点，推动外贸向优质优价、优进优出转变，努力实现外贸出口正增长。

大力振兴实体经济。牢固树立实体为本理念，努力在转型升级中做强做优实体经济。改造提升传统产业，出台优化提升汽车、电子、轻纺、食品等产业扶持政策，促进传统产业焕发新活力、形成新优势。抢抓“中国制造2025”试点示范机遇，实施企业制造装备升级和互联网化提升计划，建设30家智能工厂，全力打造中国智能制造名城。实施规上工业企业增长计划，全年力争新增规上工业企业200家以上。全面落实国家、省市支持企业发展政策，建立企业服务需求快速处置机制，协同解决企业发展难题。实施质量提升行动，推进质量强市示范城市建设，增加高质量产品和服务供给。鼓励企业弘扬“工匠精神”，发展一批名牌产品，做强一批“百年老店”。强化金融对实体经济支持，拓宽融资渠道，增加实体经济贷款份额，让更多的金融活水流向实体经济。

发展壮大民营经济。放宽市场准入，放开投资领域，推行负面清单制度，制定实施民营企业进入特许经营领域具体办法，推进一批政府与社会资本合作的重大项目，民营经济增加值占地区生产总值比重提高到46.3%。加强产权保护制度建设，落实好民营企业新技术新产品首购首用等政策。大力扶持民营企业发展，培育认定100家优秀民营企业，努力争创全国500强和江苏省100强民营企业。

（二）聚力创新突破重点，加快产业转型升级步伐。坚持把创新摆在全局发展核心位置，发挥科教优势，聚焦重点领域，突破关键环节，加速发展动力转换和产业结构调整，加快建设国家创新型城市，努力实现更有质量和效益的发展。

厚植创新发展新优势。围绕形成更多引领型发展，推动关键核心技术突破，加速高端产业集聚，在全省“一中心、一基地”建设中争当排头兵。加快建设苏南国家自主创新示范区，发挥“一区两园”核心载体作用，推动江北新区创建国家产业技术创新中心，支持徐庄软件园、新城科技园、南京软件谷争创省级高新区。加快建设科技成果转化公共服务平台，建成省技术产权交易市场，实施未来网络、第五代移动通信等重大科技专项，新建市级以上工程技术研究中心60家。大力培育创新企业，新增高新技术企业150家。推进江苏创业大街、珠江路创业大街等众创载体建设，新增众创空间20个。创建国家军民融合创新示范区，促进军民深度融合和技术双向转化。优化创新创业生态，出台降低创新创业成本、加快科技服务业发展等政策措施。实现科技服务业总收入增长15%。深入实施“创业南京”英才计划，引进18名科技顶尖专家，培育40名创新型企业家，引进630名高层次创业人才。加快建设知识产权强市，万人发明专利拥有量达42件。

加快产业转型升级。坚持高端化、智能化、绿色化、服务化、品牌化方向，加快产业结构优化提升。推动新兴产业规模发展，瞄准新一代信息技术、高端装备制造、新材料等七大类14个重点领域，着力建设智能电网、集成电路、新能源汽车等产业基地，优选培育100家新兴产业骨干企业，力争战略性新兴产业主营业务收入增长12%。推动服务业高端发展，大力发展以工业设计、检验检测、现代物流为代表的生产性服务业，以休闲旅游、文化体育、健康养老为代表的生活性服务业，以总部经济、现代金融、会展服务为代表的高端服务业，加快服务业集聚区建设，全力推动金融城二期、华侨城文化旅游综合体、世茂梦工厂、国博中心三期等服务业重点项目建设。全力打造国际软件名城，完成软件和信息服务业收入5400亿元，增长15%左右。

促进农业提质增效。大力推动农业供给侧结构性改革，不断优化提升农业产业体系、生产体系和经营体系。加快调整农业结构，推进农业生产绿色化、标准化、品牌化，增加“无公害、绿色、有机和地理标志”优质农产品供给，在全市食用农产品总量中占比达42%。深化国家现代农业示范区建设，扩大特色农业产业规模，新增高标准农田8万亩、设施农业2.5万亩，农业机械化水平达86%。细化落实承包土地“三权分置”办法，鼓励专业大户、家庭农场、农民合作社、农业龙头企业等新型经营主体发展适度规模经营，新增市级以上示范家庭农场50家，农户参加农民专业合作社比重达78%，新培育职业农民1.2万人。大力发展休闲农业，促进农业与生态、旅游、文化融合发展，休闲农业实现综合收入52亿元，接待游客超过2000万人次。实施“互联网+现代农业”行动计划，新增农业电商网店500个，农业信息化覆盖率达61%。

（三）聚焦富民力补短板，让人民生活更加幸福。自觉践行以人民为中心的发展思想，围绕“七个更”目标，强化以民生为本，以百姓感受为要，多办民生实事，持续增进民生福祉。

千方百计加快富民。实施城乡居民增收工程，制定出台改革富民、科技富民、财产富民等政策措施，落实重点人群差别化收入分配激励政策，促进技能人才、新型职业农民、有劳动能力的困难人员等八类群体致富，带动城乡居民总体增收。把创业作为富民增收之源，完善创业支持政策体系，建设创业创新平台，重点鼓励大学生、农民等群体创业，全年培育自主创业者1.3万人。把就业作为富民增收之本，坚持以产业层次提升带动收入水平提高，努力创造更多适合高素质劳动者的智力型、技术型、管理型就业岗位。深化农村土地使用制度改革，规范农民承包土地经营权流转，推进农村宅基地和农房统一登记试点，增加农民财产性收入。持续推进脱贫攻坚，对全市3.94万户低收入农户实施“一户一策”精准扶贫，着力开发三大集中连片欠发达地区，完成 30%的低收入农户和经济薄弱村脱贫任务。

着力提升社会保障水平。实施全民参保计划，城乡基本养老保险、基本医疗保险和城镇职工失业保险覆盖率保持在98%以上。加强社会保险市级统筹，稳妥推动机关事业单位养老保险制度改革，做好退休人员、特殊群体的养老保险待遇调整工作，推进城乡居民医保制度并轨。强化托底保障和“救急难”，健全最低生活保障等标准动态调整机制。加大安居保障力度，实现城市中低收入住房困难家庭应保尽保。

切实增加公共产品供给。建立完善基本公共服务清单和建设标准，以标准化推进均衡化。推动学前教育优质发展，力争在省市优质园就读幼儿比例达到87%，继续推进0—3岁婴幼儿早期发展工作；促进义务教育优质均衡发展，优质教育资源覆盖率达到90%。加快健康南京建设，深化综合医改，推进“医联体”、家庭医生制度、“智慧医疗”建设，实施医疗医保医药“三医联动”。提高居家、社区、机构养老服务水平，推动医养融合发展，新增养老床位3300张，养老机构民营化率达到80%。办好2017年世界全项目轮滑锦标赛等大型体育赛事。扎实做好工青妇、民族宗教、侨务、人防、档案、地方志、双拥共建、慈善等各项工作。

（四）大力推进城乡建设，全面提升城市功能品质。围绕东部地区重要中心城市、特大城市战略定位要求，一以贯之推进现代化国际性人文绿都建设，加大投入，加强治理，打造和彰显城市特色风貌，全面提升宜居宜业水平。

优化城市空间布局。推进“多规合一”，统筹江南与江北、城市与乡村、地上与地下、建设与管理等工作，明确人文历史、开放空间、视廊控制等要求，塑造具有历史风韵和文化特色的整体形象。加快构建以主城为核心，以江北新区、东山（空港）、仙林（汤山）为重点的南京都市区格局。推进江心洲生态科技岛、河西南部地区建设，高标准打造河西新城现代化城市中心。突出智慧、生态理念，加快南部新城建设。加大基础设施建设力度，提升溧水、高淳副城功能。打造沿明城墙、沿长江、沿秦淮河、沿历史街巷的生态人文空间，彰显城市个性，提升城市品位。有序推进城市更新，加快鼓楼滨江、铁心桥—西善桥、铁北、燕子矶等重点片区更新改造，提升老城区环境品质。

完善交通枢纽功能。优化提升空港、江海港、高铁港枢纽功能，着力构建多式联运体系。加快对外交通建设，启动实施宁马高速、宁合高速等一批改扩提升工程。大力推进过江通道建设。完善轨道交通体系，建成地铁4号线一期、宁和线一期，力争建成宁高线二期，全力推进地铁5号线、宁溧线、1号线北延和7号线工程，开工建设地铁2号线西延、6号线等工程。加快一批综合换乘枢纽建设，建成高淳站综合交通枢纽配套项目。进一步完善城区道路交通体系，完成南京南站枢纽快速环线龙西互通二期、绕城公路铁心桥立交改造工程，续建苜蓿园大街南延等工程，开工建设红山路—和燕路快速化改造、扬子江大道节点改造等重点道路。

加快城市品质提升。实施城市“净化、绿化、亮化、美化”工程，开展明城墙沿线、滨江岸线、主次干道、公园景区等环境整治，实施背街小巷、户外广告、店牌店招、露天杆线、油烟扰民等专项治理，加大公共停车场建设、交通疏堵保畅、城市景观照明提升等工作力度，提升城市整体形象。强化施工管理，最大限度降低对市民生产生活的影响。完善特大城市治理模式，推动全民参与城市治理，建立责权明确、智能高效的精细化管理新机制。深化智慧城管建设，实现数字化城管系统市域全覆盖。深入贯彻落实物业管理条例。推进垃圾分类处理设施建设，建成垃圾分类示范小区200个，城乡生活垃圾集中无害化处置率保持100%。

促进城乡统筹发展。深入推进国家新型城镇化综合试点，优化城乡公共资源配置，促进城乡融合发展。高标准规划建设22个特色小镇，打造生态园林式的新型产业社区。加快全域美丽乡村建设，建设示范村40个、特色村40个、宜居村100个、示范区200平方公里。深入开展农村环境综合整治，有效解决环境突出问题。实施农民建房指导意见，提升农民住房品质。加快城乡重大水利工程建设，基本完成石臼湖、固城湖防洪能力提升主体工程，启动八卦洲汊道整治、长江崩岸治理等一批重点工程，继续推进秦淮东河先导段建设，实施373处江河湖库防汛消险工程。

（五）坚持高起点高标准，加速推进江北新区建设。围绕“三区一平台”战略定位，聚焦科技创新、产业转型、重大基础设施建设和体制机制创新，全力打造创新策源地和重要增长极。

突出科技创新引领。把创新作为江北新区的立区之本，以创新驱动为核心，打造人才高地、创新高地、产业高地，建设引领发展的科技之城。把江北新区作为全市科技创新的重点突破区域，进一步争取国家级重点实验室、工程技术研究中心和省级研发机构向新区集聚，加快省产业技术研究院、中德智能制造研究所等一批科技创新平台建设，着力打造“扬子江创新港湾”和科技金融合作创新示范区。加快新区产业培育集聚，围绕“4+2”主导产业，大力推进台积电、上汽桥林基地等重大项目建设，重点打造集成电路、生物医药、新能源汽车等千亿级产业集群。推动江北新区服务贸易创新发展试点，打造长江经济带对外开放合作重要平台，新区服务贸易进出口总额增长16%以上。

完善基础功能设施。坚持高水平规划、高标准建设，合理布局江北新区生态空间、生产空间和生活空间。严格把握新区建筑形态，加强对天际线、山际线和滨水线的管控，提升城市品质、塑造城市风貌。加快核心区建设，开工建设中央商务区地下空间一期工程100万平方米，启动新区城市馆、南京美术馆新馆、服务贸易创新发展大厦等一批标志性、功能性项目。提升新区综合交通功能，推进地铁4号线二期、11号线一期和宁天线南延前期工作，续建长江隧道连接线快速化改造、滨江大道二期等道路工程。大力推进生态功能区建设，强化对长江沿岸湿地、老山森林公园等重点区域保护，启动青龙绿带一期等重点生态工程。加快市一中江北校区、南丁格尔国际护理学院等民生工程建设，力争早日投入使用。

强化体制机制建设。进一步理顺新区管理体制和运行机制，加大高位协调力度，推进扁平化管理，完善市场化项目运作模式，建立更具激励性的考核评价机制，加快形成统一决策、统一规划、统一推进的发展合力。深化行政管理体制改革，形成“集中高效审批、分类监管服务、综合行政执法”的治理架构，努力把江北新区打造成为审批事项最少、办事效率最高、创业创新活力最强的新区。充分借鉴上海浦东等国家级新区成功经验，推动海关、国检、海事、边检等口岸查验单位在新区增设分支机构，加快构建与国际接轨的新区运行管理模式。

（六）深化生态文明建设，打好改善生态环境攻坚战。深入践行绿色发展理念，全面实施“两减六治三提升”专项行动，着力补齐生态短板，持续改善生态环境质量。

大幅减少污染物源头排放。坚持调整产业结构与能源结构并重，源头控排与末端治理并举，煤耗与能耗双控，从源头上为生态环境减负。优化四大片区工业布局，加快烷基苯厂等重点企业搬迁，启动实施南化、南钢、梅钢等企业转型升级项目。减少煤炭消耗总量，严控新建燃煤耗煤项目，调整优化煤电布局，发展清洁能源，启动实施化工园供热一体化项目，完成协鑫发电和秦源热电等“煤改气”。减少化工铸造等落后产能，推进化工园区内46家化工企业限期整改转型关闭，全面实施化工园区外化工企业关停转迁计划，开展铸造等低端低效产能淘汰工作。推动企业清洁生产和园区循环化改造，完成清洁生产企业审核100家，实施重点节能技改项目100个。

更大力度治理环境污染。扎实推进大气污染防治行动计划，全面实施热电、水泥等重点行业废气治理和提标改造，严格执行机动车排放标准，重拳治理臭氧污染，加快推进重点行业氮氧化物减排，全力整治挥发性有机物污染，扎实做好重污染天气应对工作，持续改善空气质量，让市民享有更多蓝天。深入实施水污染防治行动计划，强化“河长制”管理，大力整治黑臭河道，统筹开展排口治理、存量管网清疏等工程，推进736个排水达标区创建，实施主城水系连通、引水补水工程，让城市的水更净、河更清。完成10个工业废水深度治理工程项目，实施城北、江心洲等污水处理厂提标改造，建成区污水处理率达到95%以上。开展河湖水库水生态修复，实施水断面达标工程，确保达到国考、省考标准。推动取水口整合，完成主城应急备用水源近期工程。全面落实土壤污染防治行动计划，实施化肥、农药零增长行动，进一步深化畜禽养殖污染防治，加强重点区域土壤分级分区管理，开展关停搬迁企业污染场地土壤修复试点。

切实加强生态环境建设。严格按照“共抓大保护、不搞大开发”的要求，实施沿江生态保护和修复工程，利用好长江岸线资源，全力推进长江生态廊道建设，让“黄金水道”持续产生“黄金效益”。大力推进省海绵城市试点片区建设，实施江北新区核心区、南部新城红花—机场片区、江心洲中部片区等一批示范项目。全面落实主体功能区规划，坚决守住城市开发边界、耕地保护和生态保护“三条红线”。深化转变土地利用方式创新试点，推动闲置土地和低效用地再开发。加强重要生态功能区保护，加快北部和南部生态涵养区建设，构筑生态安全屏障。启动新一轮城乡绿化行动，完成绿化造林2万亩，林木覆盖率达到29.7%，建成区绿化覆盖率达到44.6%。

实行更严生态保护制度。制定实施与污染物排放总量直接挂钩的财政政策，健全生态保护补偿办法，完善排污许可证、排污权有偿使用和交易等机制，用价格机制和市场机制倒逼企业转型。从严执行生态环境考核和责任追究等制度，实施领导干部自然资源资产离任审计试点，健全网格化环境监管体系，加大环境保护执法力度，坚决打击环保违法行为。

（七）推进重点领域改革，持续增强发展动力。着眼于解决发展中存在的突出矛盾和问题，着力提高改革的针对性和实效性，推动关键领域改革取得重大突破，更好释放体制机制活力。

深化行政审批制度改革。以“六个聚焦”为着力点，进一步深化行政审批制度改革。在江北新区、江宁区、溧水区、高淳区及省级以上开发区，开展相对集中行政许可权改革试点，着力推进“一枚印章管审批”。提升投资建设审批效率，加快推行多规合一、多图联审、多评合一、联合勘验、联合测绘、联合验收和区域性联合评价，最大限度地缩短办理时限。大力实施民生领域行政审批服务优化提升工程，在住房、教育、医疗等领域，深入推进办事流程简化优化和服务方式创新。

深化经济体制改革。持续推进商事制度改革，积极开展“证照分离”试点，探索“证照联办”，降低市场准入门槛。深化投融资体制改革，推动省市共建发展基金，支持跨江发展和江北新区重大基础设施建设。深化金融体制改革，发展天使、创业、产业投资，加强对重大项目、小微企业、“三农”等领域的金融支持。深化贸易流通体制改革，全面推进国家级物流标准化试点，促进贸易流通转型升级。推动国企混合所有制和多元化改革，推进国有企业资产重组和上市融资，重点做好符合条件的国有企业挂牌上市。

深化其他领域改革。推进科技与人才体制改革，推动在宁高校科技成果使用权、处置权、收益权改革试点，健全完善科技资源共享机制。推进民生领域改革，多渠道提高公共服务共建能力和共享水平。强化生态文明体制机制改革，加快推进战略环评、综合执法等改革试点工作。深入开展国家级农村改革试验区工作，继续完善农村产权交易市场，深化农村集体产权股份合作制改革。扎实做好价格市场化、户籍、文化等领域改革，确保各项改革任务落地生效。

（八）持续扩大对外开放，构筑开放发展新优势。抢抓多重国家战略叠加的宝贵机遇，推进更深层次、更高水平的双向开放，全面提升城市国际化水平。

主动融入国家战略。深度参与“一带一路”建设，加快推动南京企业“走出去”，鼓励轨道交通、电力通信、水泥装备等优势产业设立境外生产基地，开展国际产能合作，拓展市场空间。主动参与长江经济带建设，加强与长三角城市间的合作交流，推进宁杭生态经济带规划建设，深化扬子江城市群协作，推动交通基础设施互联互通，深化科技产业合作，进一步发挥东部地区重要中心城市、特大城市作用。

打造开放发展平台。加快全市开发区转型升级创新发展，全市省级以上开发区实际利用外资比重达到65%。全力推动三大枢纽经济区加速发展，高标准推进综合保税区建设，大力发展外向型产业集群，形成各具特色的对外开放基地。办好海峡两岸企业家峰会、第二届世界智能制造大会、首届全球未来网络峰会等重大活动。进一步降低外资准入门槛，强化产业链、价值链中高端招商，鼓励外资主要投向战略性新兴产业、高端制造业和现代服务业，提升利用外资质量和效益，全市实际利用外资达到36亿美元，吸引外商投资地区总部等功能性机构16家。拓展电子口岸功能，深化长三角口岸通关一体化建设，不断提高投资贸易便利化水平。

深化区域合作发展。大力推进宁镇扬一体化发展，加快先导区建设，以跨市域城际轨道交通、通信网络建设等为重点，实施一批重大项目，提升三市沿江生态共保共治、公共服务共建共享水平，推动一体化发展取得实质性突破，在宁镇扬一体化发展中发挥好带动作用。加快推动南京都市圈建设，打造南京都市圈中心城市与周边次中心城市、重要城镇之间0.5—1小时通勤圈。继续做好扶贫协作和对口支援工作。

（九）强化城市文化建设，大幅提升文化软实力。坚定文化自信，增强文化自觉，进一步繁荣发展社会主义先进文化，传承好南京城市“根”与“魂”，彰显历史文化名城魅力与风采。

提高城市文明程度。深入践行社会主义核心价值观，坚持用中国梦凝聚共识、汇聚力量，培育拓展新时期市民精神。大力弘扬红色文化，用好用活党史资源，传承“雨花英烈精神”，激发全市人民开拓奋进的强大动力。深化文明城市建设，推动志愿服务制度化，实施公共文明引导工程，使崇尚文明成为全社会的风范。繁荣发展哲学社会科学，建设具有南京特色的现代新型智库。举办世界知名创意城市“南京周”等活动，展示城市文化国际魅力。

保护传承城市文脉。进一步挖掘整合历史文化资源，加强城市整体格局风貌、历史地段、文物古迹、古镇古村和非物质文化遗产保护。推动“海上丝绸之路”南京史迹申遗和“中国明清城墙”联合申遗，打造城市国际文化名片。围绕明城墙重要节点，加强整体规划、改造、提升，彰显城市特色。实施颐和路历史街区、百子亭历史风貌区、南捕厅四期等一批历史文化保护、展示与复兴工程，启动大报恩寺遗址公园二期、科举博物馆二期、中华门城堡加固等工程，提高文化遗产保护利用水平。实施《南京非物质文化遗产保护条例》，建设国家民间文化传承示范基地。

繁荣发展文化事业。健全现代公共文化服务体系，完善市、区、街镇、村（社区）四级公共文化设施网络，标准化推进基层综合性文化服务中心建设，构建城市“15分钟文化圈”、农村“十里文化圈”。创新公共文化运行机制，推动江宁区、建邺区、全市13个街镇创建国家级、省级公共文化服务体系示范区。深化文化惠民“百千万工程”，开展送演出、送电影下乡等活动。推进全民阅读，创成首批“江苏省书香城市建设示范市”。建成艺术小学新校，完成广播电视节目无线数字化建设。实施文艺精品创作工程，设立南京艺术基金，鼓励创作更多弘扬主旋律、激发正能量、集思想性艺术性于一体的精品力作。

提升文化产业竞争力。推动文化与旅游、科技、金融及互联网等融合发展，大力发展创意设计、动漫游戏等新型文化业态，努力建设全国重要文化创意中心，文化产业增加值占地区生产总值比重提高0.3个百分点。优化文化产业空间布局，建成秦淮老城南历史文化传承创新和长江路文化休闲体验示范功能区。完善“创意南京”产业融合公共服务平台体系，积极创建“全国文化金融合作试验区”。扩大文化消费试点，增加文化有效供给，聚焦文化特色塑造，打造南京文交会、《报恩盛典》等特色文化品牌。

（十）加强社会治理创新，提升现代社会治理水平。深入实施社会治理创新工程，健全完善现代社会治理模式，切实维护社会和谐稳定。

切实夯实基层基础。进一步健全基层社会治理体系，深化街道和社区体制改革，持续推进城乡社区“减负增效”，加快街镇社区新型综合服务中心建设，城市和农村和谐社区建设合格率分别达95%和90%。全面推广“为民服务全程代理”，推动全国农村社区建设示范单位申报，促进公共管理服务向农村社区延伸。以社区居民亟需的基本公共服务为导向，培育和扶持一批提供养老、为小、调解、助困等服务的社区社会组织，促进社会组织健康有序发展。做好新一届村（居）委会成员轮训，切实增强社区班子成员能力水平。加快“全科社工”队伍建设，持续壮大社工队伍。

深化平安南京建设。坚决把牢安全发展红线，严格落实安全生产“党政同责、一岗双责、齐抓共管、失职追责”要求，深入开展重点行业领域专项整治，坚决防范遏制重特大安全生产事故发生。加强食品药品安全监管，争创国家食品安全示范城市。强化灾害监测预警和风险防控，保障城市安全高效运转。完善立体化现代化治安防控体系，严密防范、依法惩治违法犯罪活动，公众安全感保持在92%以上。

创新社会治理方式。推进“诚信南京”建设，加强各类市场主体信用信息的收集、存储和应用，加快建设覆盖全市的信用信息平台。加强信访法治化建设，开展“信访积案化解攻坚年”活动，有效预防化解社会矛盾纠纷。深入实施“七五”普法规划，扎实做好人民调解、社区矫正等工作。加强流动人口和特殊人群服务管理。深化国防动员和双拥建设，巩固双拥模范城“八连冠”成果。

三、更大力度办好民生实事

新的一年，我们强化以群众满意为导向，注重民生实事问需于民，广泛征集民意，贴近群众需求，优选出具有公益性、普惠性、示范性的35件民生实事，集中力量、集中资源、集中财力，扎实推进、务求实效，真正让人民群众看得见、摸得着、得实惠，切实增强广大市民的获得感和幸福感。

1、实施交通治堵工程。完成扬子江隧道江北连接线快速化改造等6条道路建设，实施浦口区车站后、黄姚铁路道口“平改立”，推进纬七路东进二期等5项续建工程，开建红山南路东延二期等4项工程。

2、加快过江通道建设。推进南京长江五桥建设和长江大桥公路桥维修改造，开工建设浦仪公路西段，启动和燕路过江通道建设，加快开展仙新路、建宁西路等6条过江通道前期工作。

3、方便城乡居民出行。新建公交专用道不少于30公里。新辟、优化公交线路30条以上，建设27座公交场站，出新公交站亭（牌）200个以上。完成450公里农村公路提档升级和16座农路桥梁建设。

4、扩大公共自行车覆盖面。建成玄武、鼓楼等四区公共自行车综合服务中心。推进公共自行车进小区，新增服务网点360个、自行车2.5万辆。推出公共自行车手机APP，实现主城公共自行车无卡租赁。

5、提供更多停车设施。建设公共停车场21个，新增停车泊位1.5万个，投放停车自助缴费机300台。划定非机动车停放专用区域300处。

6、实施道路环境综合整治。完成玄武大道、北京东路、中华路等22条主次干道综合整治，打造100处道路节点花境。

7、加强黑臭河道整治。完成南玉带河、珍珠河等109条黑臭河道治理，基本消除建成区河道黑臭现象。

8、改造积淹水片区（点）。完成兰家庄、观门口北村、中山门大街等35处积淹水片区（点）改造。

9、完善公共厕所配建。新建改造公厕645座，全部达到二类以上公厕标准。消除城镇范围内公共旱厕。

10、提升老旧小区居住品质。实施240个、建筑面积约500万平方米老旧小区环境整治，增加机动车停车位，设置自行车停车棚充电站，安装监控设备，加装1000部住宅电梯。

11、加大住房保障力度。新开工保障房400万平方米，竣工300万平方米。筹集建设人才安居住房20万平方米。

12、推进棚户区改造。通过征收拆迁、翻改建、完善房屋使用功能等方式，完成棚户区改造350万平方米。

13、实施老旧小区二次供水设施改造。新建、改造供水管网100公里，完成主城200处老旧小区二次供水设施改造。

14、推动既有建筑节能改造。实施绿色建筑、可再生能源建筑应用和节能改造示范工程，建成绿色建筑500万平方米。

15、提升景观亮化照明。实施秦淮河（清凉门—集庆门段）、新庄立交、中央门立交、江东路沿线、城西干道、城东干道、滨江河西段（示范段）亮化提升工程。

16、扶持高校毕业生创业就业。扶持青年大学生创业4500人。加大在宁就业创业高校毕业生租房补贴政策落实力度，简化办事流程和申办手续，降低就业创业成本，全年补贴符合条件的高校毕业生2万名以上。

17、援助困难人员就业。为就业困难人员提供职业指导培训、职业介绍、岗位推荐等援助工作，确保零就业家庭动态清零。全年政府补贴性职业培训10万人次，组织招聘会1000场，提供岗位29万个。新增残疾人就业1000人。

18、做好价格稳控工作。实施物价上涨与困难家庭临时生活补贴联动机制，推进农副产品平价商店规范化建设，确保全市稳定运营的平价商店不低于200家。

19、做好精准扶贫工作。农村年人均可支配收入9000元以下的低收入人口实现脱贫1.2万户，21个经济薄弱村、39个经济欠发达村实现脱贫。

20、提高养老保障待遇。城乡居民基础养老金最低标准从每月345元增加到380元，被征地人员老年生活困难补助每月增发45元。

21、提高医疗保险财政补助标准。老年居民财政补助标准从460元提高到500元，其他居民财政补助标准从430元提高到480元。新型农村合作医疗人均财政补助标准从620元提高到660元。

22、实施小学“弹性离校”。安排财政专项经费，为全市小学生提供延时照顾服务，缓解部分学生家长接孩子难问题。

23、改善学校办学条件。新改扩建幼儿园25所，新创建省市优质幼儿园30所。实施“中小学厕所提升工程”，新改扩建中小学厕所1万平方米以上。创建20所中小学、幼儿园“智慧校园”示范校。创建20所“园林式”校园。

24、扩大医疗资源供给。完成南部新城医疗中心建设，加快建设鼓楼医院江北医疗中心，建成市第一医院河西院区，完成溧水区中医院异地新建和栖霞区妇幼保健院建设。

25、提高基层医疗服务能力。在社区卫生服务中心开设市级特色专科15个，全市48个“医联体”全年下派医务人员400名。

26、开展健康体检服务。为40万企业退休人员提供免费健康体检。开展市民体质检测3万人次以上。

27、增设公共场所母婴室。在机场、车站、商场、公园、图书馆、医院、政务服务大厅等公共场所以及女职工较多的企业，兴建扩建120个标准化母婴室。

28、强化社区养老服务。对3A级以上社区居家养老服务中心，按等级给予综合补助。新建17个老年人日间照料中心、100个社区老年人助餐点。为75周岁以上的独居老人提供安装紧急呼叫终端补贴。为经济困难的半失能、失能老人购买居家照护服务，购买标准分别提高到400元/月和700元/月。

29、丰富群众文化生活。新建综合性公共文化服务中心250个。举办各类展览200场，组织公益演出广场行100场，公益音乐会22场，市属院团赴基层公益演出300场，公益电影放映7500场。组织全民阅读活动2000场，打造新型阅读空间20个，新建图书漂流文化驿站100个。

30、积极推动全民健身。巩固提升城市社区“10分钟体育健身圈”，更新400套全民健身工程（点）健身器材，开展各类群众性体育竞赛活动不少于2000项次。

31、开展公益科普活动。安装科普阅览屏200余台，制作播放公益科普宣传片50余集，举办“南京科协大讲堂”报告会100场、科普大学教学活动400场。

32、改善休闲旅游服务。完成主城区41个约90万平方米游园绿地新改建任务。建成南京南站旅游集散中心。全面开放已建成的滨江大道风光带公共配套设施。

33、方便市民城郊游览。开通城区旅游观光巴士专线10条以上、“美丽乡村游直通车”线路10条以上。

34、保障百姓餐桌上的安全。按照常住人口每千人4份的标准，加大对33大类食品的日常抽检力度。在7家批发市场建立食品安全快检中心，在80个农贸市场建设食品安全快检室。新创建食品生产加工小作坊示范点50家。

35、推进智慧城市惠民。拓展“我的南京”智慧城市平台的服务功能，为市民挂号就诊、公交出行、停车引导、体育健身等提供高效便捷的网上服务。推进市民卡在医疗卫生、公共交通、社会保障等领域的全面应用。拓展公安“微警务”平台功能，为市民提供治安、交管、户政、出入境等便捷服务。

民生实事是一面镜子，折射的是政府部门的为民情怀；民生实事是一道考题，考验的是全市干部的作风能力。我们将带着感情和责任，把民生实事作为各级政府最应干、必须干、马上干的事情，一件件解决好，一桩桩落实好，以实实在在的成效兑现我们的承诺。

四、高水平推进政府治理现代化

新的目标、新的任务，对政府自身建设提出新的更高要求。我们要牢固树立政治意识、大局意识、核心意识和看齐意识，全面贯彻从严治党要求，坚持把从严治党落实到从严治政之中，严肃党内政治生活，从严监督管理干部，持续深入改进作风，切实增强政府系统战斗力、公信力、执行力。

（一）依法行政，从严治政。遵循法治思维、运用法治方式，在法治的轨道上行使各项权力。依法规范权责，深入贯彻落实法治政府建设实施规划，进一步规范权力事项管理，构建互联高效的“行政权力运行网络平台”，加强权力清单动态管理和考评机制，为权力运行提供科学的法制保障。健全依法决策机制，落实重大行政决策合法性审查制度，发挥好政府法律顾问作用。完善行政执法体制，推动跨部门、跨领域基层综合执法，严格规范行政执法行为，落实行政执法责任制。强化监督机制，自觉接受市人大及其常委会法律和工作监督、市政协民主监督，切实做好建议提案办理工作，加强审计监督，主动接受群众监督和媒体监督。

（二）转变职能，高效服务。推进简政放权、放管结合、优化服务改革向纵深发展，切实转变政府职能，提高行政效能。全面推进国家“互联网+政务服务”试点，大力推广“一号申请、一窗受理、一网通办”，加快建设市、区、街联动的政务服务网，并延伸至社区（村），着力打通群众办事“最后一米”。坚持“亲”的服务，坚守“清”的底线，努力构建健康新型的政商关系。建设好投资建设代办服务中心、企业服务中心，完善项目推进和工作协调机制，积极主动为企业提供周到服务，切实提高行政服务效能，认真当好推动经济发展的“店小二”。深入推进全面政务公开，加强政策解读，做好重大政策第三方评估工作，探索建立信息化决策支持系统，努力打造阳光政府。

（三）敢于担当，积极作为。大力发扬苦干实干的作风，为南京发展担当、为全市人民尽责，以作风建设的更大实效推动形成改革发展的更大成效。坚持以严明的责任抓落实，真正在干事业上用心思、抓落实上想办法、求实效上下功夫，围绕经济社会发展目标，细化任务分解，形成上下贯通、层层负责的主体责任链，确保各项决策部署落实到位。坚持以铁的纪律抓落实，优化完善绩效考核体系，强化严格的督查问责机制，做到抓铁有痕、踏石留印。坚持以优良的作风抓落实，做到真抓实干、马上就办，从实处着眼、往实处用劲，以“钉钉子”精神推动各项工作落实。

（四）改进作风，勤政廉政。持之以恒正风肃纪，坚定不移反腐倡廉，倾力塑造风清气正的政府良好形象。坚持把纪律和规矩挺在前面，坚决贯彻党内政治生活准则和党内监督条例，认真落实中央八项规定，坚决防止和纠正各种“四风”新问题。持续推进反腐倡廉，从严落实“一岗双责”，聚焦侵犯群众切身利益的“微腐败”，盯紧征地拆迁、扶贫攻坚、惠民利民等重点领域，做到有腐必惩、有贪必肃。严格公务员队伍管理，强化平时考核，加大力度整治懒政怠政、为官不为，旗帜鲜明地为改革创新者撑腰鼓劲，对工作成效明显的地区加大激励支持力度，让广大干部愿干事、敢干事、能干成事。

各位代表，做好今年的工作，意义重大，任务艰巨，使命光荣。让我们更加紧密团结在以习近平同志为核心的党中央周围，在省委省政府和市委的坚强领导下，不忘初心，继续前进，勇于担当，积极作为，以推进“一个高水平建成、六个显著”，建设“强富美高”新南京的新业绩，迎接党的十九大胜利召开！
